# The extreme depletion of ionospheric electron density and its hemispheric asymmetry during the May 2024 storm

**DOI:** 10.1093/nsr/nwaf307

**Published:** 2025-08-01

**Authors:** Yanhong Chen, Ercha Aa, Tianjiao Yuan, Shunrong Zhang, Hua Shen, Xinan Yue, Heng Xu, Siwei Liu, Xin Wang, Wengeng Huang, Hui Li, Bingxian Luo, Qinghe Zhang, Chi Wang

**Affiliations:** State Key Laboratory of Solar Activity and Space Weather, National Space Science Center, Chinese Academy of Sciences, Beijing 100190, China; State Key Laboratory of Solar Activity and Space Weather, National Space Science Center, Chinese Academy of Sciences, Beijing 100190, China; State Key Laboratory of Solar Activity and Space Weather, National Space Science Center, Chinese Academy of Sciences, Beijing 100190, China; College of Earth and Planetary Sciences, University of Chinese Academy of Sciences, Beijing 101408, China; Haystack Observatory, Massachusetts Institute of Technology, Westford, MA 01886, USA; State Key Laboratory of Solar Activity and Space Weather, National Space Science Center, Chinese Academy of Sciences, Beijing 100190, China; College of Earth and Planetary Sciences, University of Chinese Academy of Sciences, Beijing 101408, China; Key Laboratory of Earth and Planetary Physics, Institute of Geology and Geophysics, Chinese Academy of Sciences, Beijing 100029, China; State Key Laboratory of Solar Activity and Space Weather, National Space Science Center, Chinese Academy of Sciences, Beijing 100190, China; College of Earth and Planetary Sciences, University of Chinese Academy of Sciences, Beijing 101408, China; State Key Laboratory of Solar Activity and Space Weather, National Space Science Center, Chinese Academy of Sciences, Beijing 100190, China; College of Earth and Planetary Sciences, University of Chinese Academy of Sciences, Beijing 101408, China; State Key Laboratory of Solar Activity and Space Weather, National Space Science Center, Chinese Academy of Sciences, Beijing 100190, China; State Key Laboratory of Solar Activity and Space Weather, National Space Science Center, Chinese Academy of Sciences, Beijing 100190, China; State Key Laboratory of Solar Activity and Space Weather, National Space Science Center, Chinese Academy of Sciences, Beijing 100190, China; College of Earth and Planetary Sciences, University of Chinese Academy of Sciences, Beijing 101408, China; State Key Laboratory of Solar Activity and Space Weather, National Space Science Center, Chinese Academy of Sciences, Beijing 100190, China; College of Earth and Planetary Sciences, University of Chinese Academy of Sciences, Beijing 101408, China; State Key Laboratory of Solar Activity and Space Weather, National Space Science Center, Chinese Academy of Sciences, Beijing 100190, China; College of Earth and Planetary Sciences, University of Chinese Academy of Sciences, Beijing 101408, China; State Key Laboratory of Solar Activity and Space Weather, National Space Science Center, Chinese Academy of Sciences, Beijing 100190, China; College of Earth and Planetary Sciences, University of Chinese Academy of Sciences, Beijing 101408, China

**Keywords:** total electron content, electron density depletion, hemispheric asymmetry, disturbed electric field, neutral composition

## Abstract

The Earth's ionosphere plays a critical role in radio wave transmission, reflection, and scattering, directly affecting communication, navigation, and positioning systems. However, the comprehensive impacts of space weather remain to be fully established in cases where the ionosphere experiences strong disturbances during geomagnetic storms. We reported unprecedented observational evidence of extreme ionospheric electron density depletion and its hemispheric asymmetry during the May 10–12, 2024 super geomagnetic storm, utilizing multi-instrument ground-based and spaceborne *in-situ* observations. The ionospheric electron density significantly decreased, with a maximum reduction of 98% over the whole northern hemisphere for more than 2 days, causing backscatter echo failures in multiple ionosondes within the Chinese Meridian Project (CMP) monitoring network. In contrast, mid-to-low latitude regions in the southern hemisphere exhibited electron density enhancements. Thermosphere-Ionosphere-Electrodynamics General Circulation Model (TIEGCM) simulations demonstrated strong consistency with northern hemispheric observations. The vertical drift and the column integrated ratio of O and N_2_ (ΣO/N_2_) from observations and simulations indicated the deep reduction of total electron content (TEC) mainly generated by severe ion recombination associated with neutral composition changes that interacted with the disturbed electric field. The summer to winter neutral wind and asymmetry of O/N₂ were possibly responsible for the asymmetry in electron density between the northern and southern hemispheres. These results advance understanding of ionospheric storm physics by establishing causal links between magnetosphere-thermosphere coupling processes and extreme electron density variations, while providing critical observational constraints for space weather model refinement.

## Teaser

An extreme depletion of ionospheric electron density was observed during the super geomagnetic storm on May 2024, resulting in severe HF radio blackout. The reduction in electron density exhibits a significant asymmetry between the northern and southern hemispheres. Numerical simulations and multi-instrument observations confirm that the changes in neutral composition, interacting with disturbed electric fields, are primarily responsible for these phenomena.

## INTRODUCTION

The Earth's ionosphere is widely used in radio communication, navigation, positioning, and over-the-horizon radar detection. Changes in the ionospheric state can directly disturb or even interrupt the propagation of radio waves. The ionospheric state is mainly affected by solar and geomagnetic activities, which have been studied for decades. During a geomagnetic storm, the ionosphere is often found to be disturbed regionally and sometimes globally on multiple scales due to the intrusion of solar wind energy and mass into the Earth's polar regions. These disturbances can manifest as increases or decreases in ionospheric electron density, known as ionospheric positive or negative storms, respectively. It is generally accepted that the ionospheric negative storm is primarily the result of storm-time neutral composition changes from high to low latitudes [1–4]. The causes of the positive storm are much more complicated, with proposed mechanisms including particle precipitation, equatorward neutral winds, penetration of electric fields, neutral composition changes, and traveling atmospheric disturbances [5–18]. The detailed processes of the ionospheric response to a specific storm are still not fully understood.

The super geomagnetic storm that occurred on May 10–12, 2024 (referred to as the Mother's Day Storm) was the most intense one in the past two decades, which provides an excellent opportunity to understand the mechanism of the ionospheric response to extreme geomagnetic storms, especially as there are more abundant observations available nowadays. Zhang *et al.* [19] analyzed the ionospheric positive and negative disturbances over the Asian–Australian and American sectors during this super storm. Aa *et al.* [20] investigated significant midlatitude plasma density peaks and dual hemisphere SED during the storm. Singh *et al.* [21] reported the super plasma fountain and induced enhancement of electron density over the Peruvian sector. Jin *et al.* [22] demonstrated a significant plasma density depletion from high‐ to mid-latitudes from Swarm *in-situ* observations. In addition, some studies have focused on the phenomenon of abnormally night-time enhancement of plasma density on May 11, 2024 [23,24].

In this study, the ionospheric response to the super storm on May 10–12, 2024 in the Chinese region was first reported by multi-instrument observations from a comprehensive and large coverage ground-based space environment monitoring network, namely the Chinese Meridian Project (CMP) [3,25]. The global response was then investigated and simulated utilizing the Thermosphere-Ionosphere-Electrodynamics General Circulation model (TIEGCM). We look into the asymmetry between the northern and southern hemispheres and the underlying possible mechanism by integrating the observational data from the Global-scale Observations of the Limb and Disk (GOLD), Swarm and Defense Meteorological Satellite Program (DMSP) satellites.

## RESULTS

### Interplanetary and geomagnetic conditions during May 10–12, 2024

**Figure 1.**
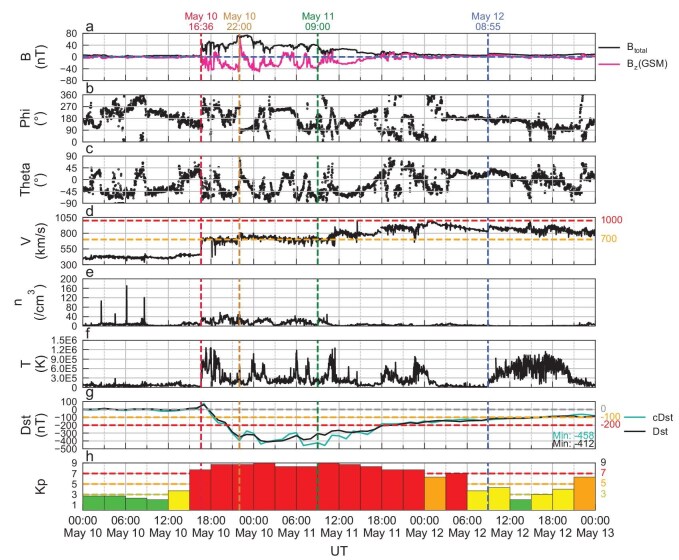
Solar wind, interplanetary environment, and geomagnetic activity indices from May 10 to 12, 2024. (a) The north south (Bz) component of interplanetary magnetic field (IMF). (b) The longitude of IMF in GSM coordinates. (c) The latitude of IMF in GSM coordinates. (d) Solar wind velocity. (e) Solar wind density. (f) Ion temperature. (g) Dst and cDst indices. (h) Kp index.

In May 2024, the solar F10.7 index increased from 135 sfu (solar flux unit, 1 sfu = 10^−22^ W ·m^−2^ ·Hz^−1^) on May 1 to 233 sfu on May 9, and remained above 214 sfu during May 10–13, indicating solar activity reached a high level (F10.7 >200 sfu). A total of 12 X-class flares erupted from the active region AR3664, accompanied by multiple coronal mass ejections (CMEs). During May 10–11, multiple CMEs reached Earth, driving multiple distinct interplanetary shocks, which were observed by the ACE satellite (shown by vertical dashed lines in Fig. 1). The southward component of the interplanetary magnetic field (IMF Bz) reached a minimum of −50 nT at 22:00UT on May 10, and the solar wind speed rose to a maximum of ∼990 km/s on May 12. The combined effect of these consecutive CMEs triggered severe geomagnetic disturbances, and the geomagnetic Kp index reached the strongest level of a geomagnetic storm (Kp = 9). The geomagnetic Dst index dropped to −412 nT at 03:00UT on May 11, setting a new record of geomagnetic intensity for the past 20 years (since November 20, 2003 with the minimum Dst index −422 nT). The cDst, an index in the Chinese region calculated from the geomagnetic data of the Chinese Meridian Project (CMP) [26], is consistent with a Dst minimum of −458 nT, meaning stronger disturbance of the geomagnetic field in the Chinese region.

### The extreme depletion of ionospheric electron density in the Chinese region and the resultant radio wave disruption

Ionospheric instruments of the CMP conducted observations during this event. Total electron content (TEC), as the total number of free electrons in a column of the ionosphere, can be estimated from dual-frequency Global Navigation Satellite System (GNSS) observations. Then the TEC maps in Chinese and adjacent regions (70–140°E, 15–55°N) are generated based on assimilation methods [27]. The ionosonde observations from 14 stations are also used in this study. In addition, the Incoherent Scatter Radar in Sanya (SYISR) (109.63°E, 18.35°N), one of the key instruments in CMP [28–31], provides altitude-resolved electron density and ion drift observations during the storm event.

**Figure 2.**
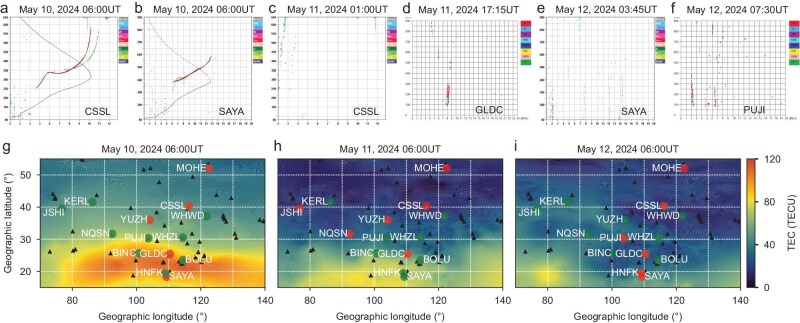
The TEC and radio wave interruptions observed in the Chinese region. (a, b) Ionogram at 06:00UT on May 10, 2024 in CSSL station (116.20°E, 40.30°N) and SAYA station (109.63°E, 18.35°N). (c, d) Ionogram interruption at 01:00UT in CSSL station and at 17:15UT in GLDC station (110.35°E, 25.35°N) on May 11, 2024. (e, f) Ionogram interruption at 03:45UT in SAYA station, and at 07:30UT in PUJI station (103.62°E, 30.31°N) on May 12, 2024. (g–i) The TEC at 06:00UT in the Chinese region on May 10–12, 2024. The red dots indicate the positions of the ionosonde stations that experienced ionogram interruptions due to ionospheric negative effects during the whole day, while the green dots are the stations with no interruptions. The small black triangles show the positions of GNSS receivers.

Figure 2g–i presents the TEC maps in the Chinese region at 06:00UT on May 10–12, 2024. The ionospheric TEC exhibited pronounced reduction (negative response) on May 11, with TEC decreasing significantly from high to low latitudes. On May 12, the TEC reduction continued and was particularly intensified in low-latitude regions. TEC between 15° and 30°N on May 10 (Fig. 2g) was about 100TECU in the daytime, whereas it decreased to 10∼20TECU on May 11–12. The TEC above 30°N on May 11–12 reduced to <10TECU (sometimes only 1–2TECU). The northern crest of the Equatorial Ionization Anomaly (EIA) was observed to be normal on May 10, while it weakened on May 11 and almost disappeared on May 12.

Figure S1 displays TEC and its deviation over the geographical latitude in the longitudes of 100°E and 120°E. The negative ionospheric disturbance occurred at high and middle latitudes on the morning of May 11 (before 12:00 Beijing time), while TEC at low latitudes initially showed a positive disturbance. Then the negative disturbance gradually expanded to lower latitudes, replacing the earlier positive response in the afternoon. The negative response in the whole of China lasted more than 2 days during the events, except for the short period of positive response after midnight on May 11 and May 12 when the electron density was normally very low. The absolute TEC difference (ΔTEC) was ∼20–30TECU at middle to high latitudes and 40–80TECU at low latitudes, respectively. The spread of TEC depletions from high to low latitudes with time suggests that changes in the neutral composition may be the major factor of the daytime negative disturbance. The Joule heating around the auroral region during the storm led to the upwelling of molecular rich air (N_2_, O_2_) to higher altitudes at high latitudes. This molecular rich air was then transported toward lower latitudes by equatorward neutral wind circulation. Thus, the ionospheric recombination rate was increased because of the decrease of the neutral composition ratio (O/N_2_), leading to the negative response of ionospheric TEC [32–34]. Figure S2 displays the observations of the column integrated ratio of O and N_2_ (ΣO/N_2_) from the Global Ultraviolet Imager (GUVI) instrument onboard the TIMED satellite during this storm event, with the local time at ∼08:30 [35]. The equatorward spread of the composition reduction zone (O/N_2_ decrease) was significantly observed on May 11, proving that neutral composition was the main cause of the TEC depletion from high- to mid-latitudes.

The magnitude of ionospheric electron density reduction was so extreme during this geomagnetic storm event that it significantly disrupted high frequency (HF) radio wave propagation. By analyzing the sequential variation of ionograms of 14 ionospheric digital ionosondes in the CMP, we classified a complete loss of signal as interruption after an obvious ionospheric negative effect, eliminating the influence of absorption caused by flares. The disappearance of ionosonde echoes caused by intense solar flares corresponds closely to the occurrence and duration of the flares. For those instances that are not accompanied by flares or persist longer than the duration of simultaneous flares, we propose they are likely caused by ionospheric negative disturbances. By analyzing the subsequent ionograms, we identified a specific recovery pattern in ionograms as the ionospheric negative storm subsides, in which echoes first reappear in the upper-left corner and gradually shift toward lower altitudes and higher frequencies. This pattern suggests that these interruptions should be attributed to ionospheric negative storms rather than flares. Figure 2g–i shows the ionosonde stations’ positions in which the ionograms are normal (green dots) or experience the interruption (red dots) during May 10–12, 2024. Figure 2a and 2b display examples of the normal ionograms, while Fig. 2c–2f show the ionogram interruptions. After the storm’s sudden commencement (∼17:00UT on May 10), the ionogram interruptions were observed at 5–6 stations on May 10, and also on May 11–12, 2024, covering the middle and low latitudes. The intense reduction in the frequencies of echoes indicated that the ionization densities decreased sharply, making them insufficient to reflect the signals from ionosondes.

**Figure 3.**
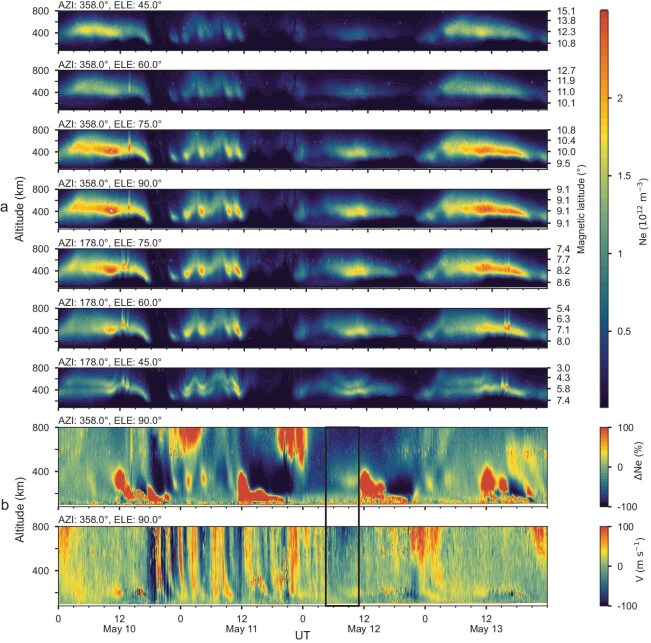
The observation of electron density, ion drift velocity from SYISR (109.63°E, 18.35°N). (a) The electron density profile in the south-north direction with different elevations on 10–13 May, 2024. (b) The electron density relative difference (upper panel) and line-of-sight ion velocity in zenith mode (bottom panel). The black box highlighted the periods of westward disturbance dynamo electric field (DDEF).

Figure 3a shows the electron density profiles in SYISR as a function of altitude along the radar beams with different elevation but the same northward azimuth on May 10–13, 2024. Figure 3b presents the change in electron density (ΔNe) in zenith mode relative to the observation on May 14, 2024, as there were no full day's observations on May 8–9, 2024. The electron density depletions were observed throughout the whole F-region altitudes, with a notable decrease in the F2 peak range of 300–600 km. The electron density at night on May 11 decreased by three orders of magnitude, with a maximum reduction of 99% (Fig. 3b). Moreover, the modes of electron density depletion were significantly different between May 11 and 12, 2024. The electron density near the peak region exhibited a fluctuating pattern on May 11, characterized by rapid decrease followed by recoveries, with this process being repeated. This wavelike structure is a manifestation of the large-scale travelling ionospheric disturbance (LSTID) [28]. The vertical drift (Fig. 3b) displayed multiple uplift and downward transitions, suggesting the existence of eastward and westward penetration electric fields (PEFs) associated with southward-to-northward turnings of the IMF Bz component [34]. The electron density on May 12 shows an overall decline without a distinct fluctuating pattern, and the ion velocity showed a downward vertical drift during 05:00–10:00UT in the afternoon, indicating the development of a westward disturbed electric field, which can suppress the formation of EIA and subsequently induce the ionospheric negative response. Therefore, the disappearance of EIA may be the result of combined effects of disturbed electric field and changes in neutral composition.

### Significant hemispheric asymmetric of global ionospheric negative response on May 10–13, 2024

To further expand the scope from the Chinese region to a global scale, the global TEC data sourced from the Madrigal database are employed to explore the storm-time ionospheric responses. The Madrigal database, maintained by the Massachusetts Institute of Technology Haystack Observatory, offers high-resolution TEC data products from a dense network of over 5000 GNSS receivers distributed globally. The gridded TEC data is distributed to the scientific community with a spatio-temporal resolution of 1° (longitude) by 1° (latitude) and 5 min [36,37].

**Figure 4.**
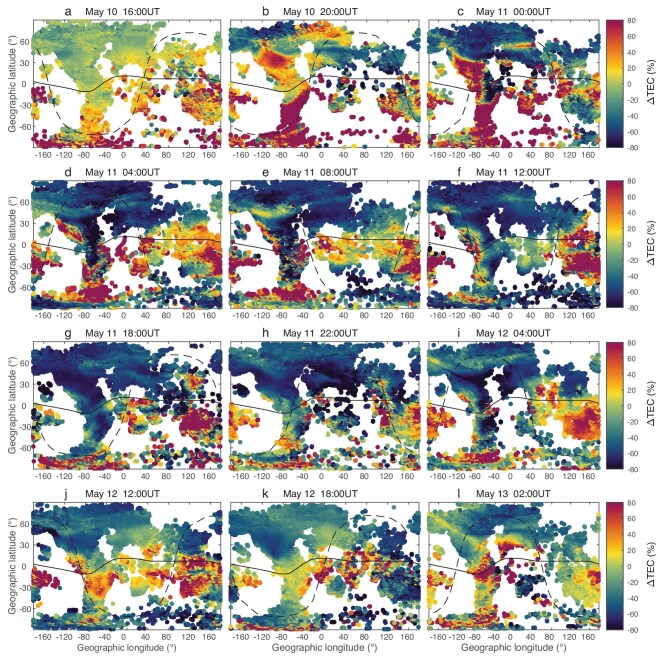
The global map of relative TEC difference during May 10–13, 2024. The black line is the magnetic dip equator while the black dash curve denotes the sunrise terminator at 400 km altitude. (a, b) The $\Delta$TEC maps at 16:00UT and 20:00UT on May 10, 2024. (c–h) The $\Delta$TEC maps at 00:00UT, 04:00UT, 08:00UT, 12:00UT, 18:00UT, and 22:00UT on May 11, 2024, respectively. (i–k) The $\Delta$TEC maps at 04:00UT, 12:00UT, 18:00UT, and 22:00UT on May 12, 2024, respectively. (l) The $\Delta$TEC map at 02:00UT on May 13, 2024.

Figure 4 shows the map of global relative TEC difference at several fixed time points during May 10–13, 2024. Although TEC data were absent in many regions, the response of the global ionosphere during this geomagnetic storm can still be clearly observed. Many features of ionospheric response can be obtained from Fig. 4, which have been reported in recent studies [19–24,32,^38^,^39^]. After the initial phase of the geomagnetic storm, it was evident that in the American sector (longitude 40°W∼120°W), the TEC enhanced significantly (Fig. 4b and 4c) from high to low latitudes in the southern hemisphere and mainly middle to low latitudes in the northern hemisphere. There was a latitudinally narrow enhanced region in the northern hemisphere extending from mid-latitude 40°N toward west and higher latitudes (Fig. 4b and 4c), with longitude ∼120°W. This is the storm-enhanced density (SED) phenomenon of the ionosphere [34,36], which was also reported for this storm event in Aa *et al.* [20]. In Fig. 4c and 4d, a large-scale TID was observed in the American sector, potentially driven by neutral wind disturbances and storm-generated atmospheric gravity waves (AGWs) [32]. In addition, localized TEC enhancement at the middle latitude in the Asian sector on May 11, as shown in Fig. S1, was also observed in Madrigal TEC and showed significant westward and equatorward movement (Fig. 4g–i), attracting the attention of many researchers [19,39]. TEC enhancement in equatorial and low-latitude regions in the American–Pacific–Asian sectors existed from 16:00UT on May 10 to 12:00UT on May 12, 2024, as analyzed in Aa *et al.* [20].

In this paper, we mainly look into the global ionospheric negative responses and the difference between the northern and southern hemispheres, which have not been comprehensively analyzed so far.

The negative ionospheric storms began to appear in high latitudes after the onset of a geomagnetic storm. The extreme electron density depletion then extended from high to lower latitudes, and gradually covered almost the whole northern hemisphere during the main and recovery time. Specifically, in North America, when the geomagnetic storm occurred during the daytime, a strong ionospheric positive storm was observed in equatorial and low-latitude regions, while TEC depletions occurred at high latitude regions simultaneously. The depletions extended toward lower latitudes and even crossed the equator on May 11–12. In the European region, the TEC depletion was significant at high to low latitudes with a significant TID occurring on May 10. In the Asian sector, as analyzed in the Chinese region, extreme TEC depletion was also observed during the night of May 10, 2024. On May 11, the positive response occurred at equatorial and low-latitude regions in the daytime of European and Asian sectors, then the response became negative after 04:00UT and persisted until May 13, except for localized positive disturbances in low-latitude regions. The maximum TEC depletion could be 100TECU, with the strongest reductions observed in the low latitudes of America, where the relative decrease could reach 98%. However, the TEC response in the southern hemisphere was not fully consistent with that in the northern hemisphere. The decrease of electron density at high latitudes of the southern hemisphere started much later, showing an obvious asymmetric change compared with that in the northern hemisphere. The TEC negative response was significant in South America, while it only located in middle to high latitudes in other sectors of the southern hemisphere.

The interhemispheric asymmetry was also observed in *in-situ* measurements. Figure S3 shows the observations of electron densities from Swarm A, Swarm B, and plasma density from DMSP, with the altitudes of the three satellites being 470, 520, and 840 km, respectively. Since the orbit positions of the satellites do not completely coincide, the satellites’ data were binned 2^o^ in latitude, 20^o^ in longitude and 0.4 hours in UT. Then the deviation of electron density or plasma density was calculated by subtracting the values of geomagnetically quiet days (from May 7–9, 2024). On May 10, the electron density from Swarm satellites indicated a significant expansion of the EIA from low latitudes to middle latitudes, leaving an electron density hole at the equatorial region. This is the effect of a super-fountain which is related to the prompt penetration electric field (PPEF) induced by the southward Bz [40]. A pronounced reduction in electron density was observed extending from high to low latitudes in the northern hemisphere, persisting throughout the entire recovery phase. In contrast, such reductions were rarely detected in the southern hemisphere, but enhancements were observed instead, which were more pronounced in Swarm satellites. The plasma density from DMSP also presented a significant enhancement from low to middle latitudes on May 10, reaching higher latitudes in the southern hemisphere, while there was no depletion in the equatorial region. The differential plasma density in Fig. S3f clearly exhibits very obvious inconsistency between the northern and southern hemispheres, which was consistent with results of Swarm A and B.

### The simulations from TIEGCM over the American, European, and Asian-Australian Sectors

The observation of GUVI O/N_2_ and the vertical drift in SYISR can, to some extent, explain the variations of TEC in the Chinese region, while further verification and interpretation are required to understand ionospheric response based on global observations. In this paper, model simulation was carried out using the Thermosphere-Ionosphere-Electrodynamics General Circulation model (TIEGCM) developed by the National Center for Atmospheric Research (NCAR) [41–43]. The TIEGCM is an all-encompassing, first-principles-based, three-dimensional, and nonlinear model that depicts the coupled thermosphere and ionosphere system. Notably, it incorporates a self-consistent solution for the low-latitude dynamo electric field [34]. At each time step, the model solves the Eulerian equations of continuity, momentum, and energy for both neutral and ion species. It adopts pressure surfaces as the vertical coordinate and covers an altitude range from ∼95 to 1000 kilometers. It has a high resolution of 2.5° × 2.5° in both latitude and longitude. In this study, the TIEGCM simulation was employed with the Weimer high-latitude convection model based on the CCMC platform (https://ccmc.gsfc.nasa.gov/models).

**Figure 5.**
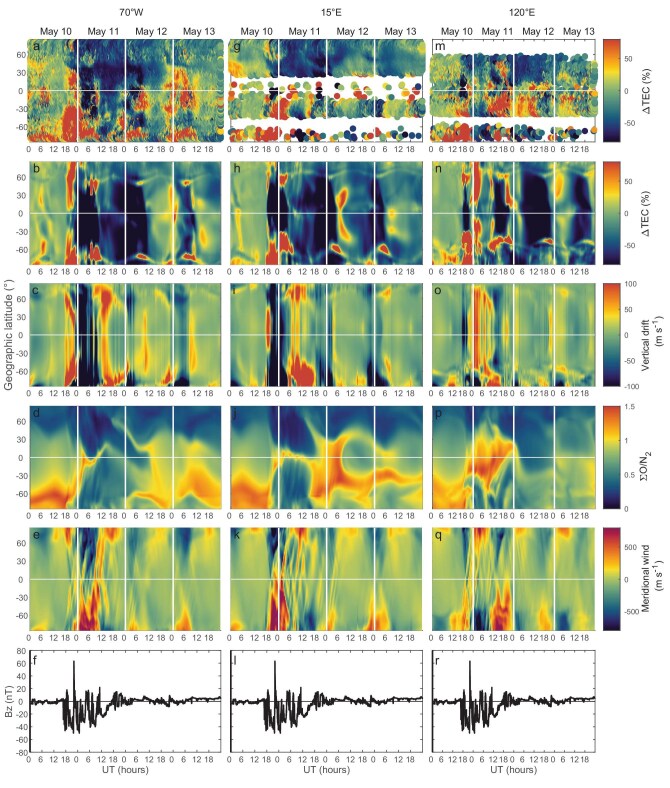
Observation of TEC relative difference, simulation of TEC relative difference, vertical drifts (upward positive), O/N_2_, and meridional winds (northward positive) at pressure level 2 (∼300 km) as a function of universal time and geographic latitude during May 10–13, 2024. (a) GNSS-TEC relative difference at 70°W. (b) TIEGCM-TEC relative difference at 70°W. (c) TIEGCM simulation of vertical drift at 70°W. (d) TIEGCM simulation of ∑O/N_2_ at 70°W. (e) TIEGCM simulation of meridional wind at 70°W. (f) The north south (Bz) component of IMF. (g) GNSS-TEC relative difference at 15°E. (h) TIEGCM-TEC relative difference at 15°E. (i) TIEGCM simulation of vertical drift at 15°E. (j) TIEGCM simulation of ∑O/N_2_ at 15°E. (k) TIEGCM simulation of meridional wind at 15°E. (l) The north south (Bz) component of IMF. (m) GNSS-TEC relative difference at 120°E. (n) TIEGCM-TEC relative difference at 120°E. (o) TIEGCM simulation of vertical drift at 120°E. (p) TIEGCM simulation of ∑O/N_2_ at 120°E. (q) TIEGCM simulation of meridional wind at 120°E. (r) The north south (Bz) component of IMF.

Figure 5 presents the TEC variations from Madrigal dataset and TIEGCM outputs of TEC difference, the vertical plasma drifts (E × B, positive upward), ΣO/N_2_, and the meridional winds (positive northward) for longitude 70°W, 15°E, and 120°E at pressure Level 2 (∼300 km). It is evident that the extreme TEC depletion was simulated in TIEGCM, from high to low latitudes during May 11–12, suggesting that the simulation mostly reflects the physical processes despite some differences from the observations. The simulated TEC depletions exhibit approximate symmetry in distribution and duration, which will be discussed later.

In the American sector (70°W), the EIA enhancement and expansion with a depletion in equatorial region from TIEGCM after 18:00UT on May 10 (Fig. 5b) were consistent with the result from observations (Fig. 5a). The model indicated the TEC enhancements covering high to low latitudes, but the observations of positive TEC disturbance in the northern hemisphere were only located in middle to low latitudes. On May 11–12, the modeled TEC depletions were in agreement with those in GNSS-TEC in most regions, with some differences from 00:00–12:00UT at the southern middle and low latitude regions. The simulation of E × B vertical drift (Fig. 5c) indicated the daytime upward drift and night-time downward drift, with rapid fluctuation on May 10–11, resulting from PEF associated the southward Bz (Fig. 5f). The upward drift enhanced electron density by moving the electron to higher latitude with lower recombination, while downward drift conversely reduced electron density. The enhanced vertical drift on May 10 simulated by TIEGCM verified the cause of the super plasma fountain and transform of electron density to higher latitudes [22]. The significant downward drift (Fig. 5c) during night-time was one of the factors contributing to the extreme TEC depletion. Another key factor is the neutral compositions’ disturbance from high to low latitudes (Fig. 5d). During the summer season, the global thermospheric circulation exhibits a summer-to-winter hemispheric transport pattern. In the quiet period (prior to 16:00UT on May 10), this circulation configuration resulted in a lower ΣO/N_2_ ratio observed in the high-latitude regions of the northern hemisphere compared to that in the southern hemisphere (Fig. 5d). Following the onset of the geomagnetic storm, equatorward neutral winds, induced by the higher temperature due to the Joule heating in high latitudes, facilitated the latitudinal transport of O/N_2_ from both hemispheres toward lower latitudes. Simultaneously, the storm-driven circulation triggered atmospheric upwelling at high latitudes, leading to a depletion of O/N_2_ in these regions. This modified O/N_2_ distribution propagated equatorward under the influence of the neutral wind and significantly reduced the electron density. Figure 5d and 5e indicates this progress clearly. The depletion of ΣO/N_2_ in both hemispheres moved equatorward with the enhanced neutral wind on May 10–11, even crossing the equatorial zone. This process significantly reduced TEC from high to low latitudes.

For the European–African sector (15°E), there was a significant TEC enhancement observed from high to low latitudes in the southern hemisphere from 18:00UT on May 10 to 08:00UT on May 11 (Fig. 5g). The simulation agreed well with the observations at high to middle latitudes, but showed depletion at low-latitude regions (Fig. 5h). During this period, the equatorial meridional wind became stronger (Fig. 5k) with the onset of the storm, which may be the main cause of the enhancement during this time. Similarly, a long-lasting negative ionospheric response was observed by GNSS-TEC (Fig. 5g) and simulated by TIEGCM (Fig. 5h) from the initial phase to recovery phase. The significant enhancement with downward drift (Fig. 5i) during the night was possibly related to TEC depletion at low latitudes. Similar to the pattern observed in the American sector, theΣO/N_2_ (Fig. 5j) disturbance moved to low-latitude regions on May 10–11, which was related to the TEC reduction in both southern and northern hemispheres. After 18:00UT on May 11, the ΣO/N_2_ in the southern hemisphere showed no reduction.

In the case of the Asian–Australian region (120°E), during the period after the storm onset, between 16:00UT and 00:00UT on May 10, 2024, significant TEC depletion (Fig. 5m) in low-latitude regions were well simulated by TIEGCM (Fig. 5n), with enhanced downward drift (Fig. 5o). At high latitudes, the simulation indicated a significant enhancement in TEC, which was consistent with the observations in the southern hemisphere. On May 11, the observation presented the TEC depletion in high latitudes and enhancement in middle to low latitudes before 12:00UT and then the depletion extended to low-latitude regions in the northern hemisphere, while in the southern hemisphere, the TEC enhancement after 12:00UT was significant. It is notable that the localized TEC enhancement region in middle to low latitudes in the northern hemisphere from 17:00UT to 21:00UT, as shown in Figs 3 and 5, was simulated well by our model. On May 12, the observation indicted a TEC increase in the low-latitude regions of the southern hemisphere, while it was mainly characterized by a decrease in the northern hemisphere. However, the simulation showed substantial TEC reductions in both hemispheres. The ΣO/N_2_ showed an enhancement in the equatorial region on May 11 and a reduction on May 12–13, especially in the northern hemisphere.

## DISCUSSION AND CONCLUSION

**Figure 6.**
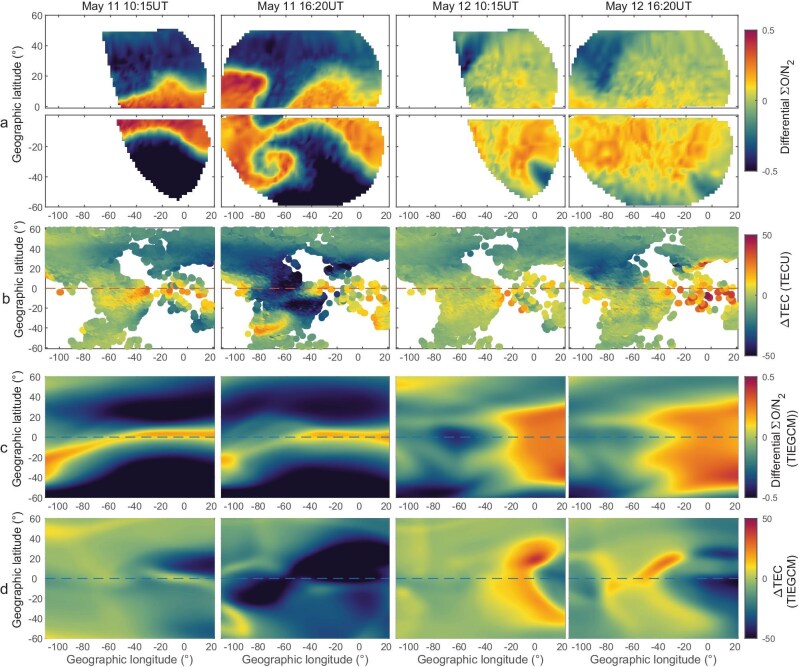
The observation and simulation of ΣO/N_2_ and TEC difference at two scan periods of GOLD on May 10–12, 2024. (a) The GOLD ΣO/N_2_ observations at ∼10:15 and 16:20 on May 11–12, 2024. The difference is obtained by subtracting the observation in the same scan period on May 9, 2024. (b) The ΔTEC from the Madrigal database obtained by subtracting the median value during May 7–9, 2024. (c) The simulated differential ΣO/N_2_ at 10:15 and 16:20 on May 11–12, 2024 obtained by subtracting the simulations on May 9, 2024. (d) Simulated ΔTEC at 10:15 and 16:20 on May 11–12, 2024 obtained by subtracting the TEC simulations on May 7–9, 2024.

The negative storm during the May 2024 storm actually lasted for more than 2 days, with the largest TEC depletion of ∼100TECU in low-latitude regions, accompanied by the disappearance of the EIA. The global ionospheric electron density revealed a very obvious characteristic of asymmetry between the northern and southern hemispheres. In the mid- and low-latitude regions of the southern hemisphere, there were positive disturbances at different time periods on May 11–12, 2024. The simulation indicated the ratio of O/N₂ is significantly different between the northern and southern hemispheres, related to the global circulation from the summer hemisphere to the winter hemisphere, which implied that changes in composition and neutral winds may be responsible for ionospheric hemispheric asymmetry during the superstorm. The observations of GUVI did not cover the southern hemisphere during this event (Fig. S2). Global-scale Observations of the Limb and Disk (GOLD) satellite, which is located in geostationary orbit at 47.5°W, provides the observations of O/N_2_ column density ratio between 70°N and 70°S and 30°E–127°W. The GOLD imagers scanned the Earth's full disk from 06:10 to 23:05UT in the far ultraviolet (FUV) wavelengths in the range of ∼134–162 nm. The retrieval algorithm of ΣO/N_2_ has been described in detail in Eastes *et al.* [44]. Figure 6 presents the GOLD's differential column density ratioΣO/N_2_ on 11–12 May, during four scanning periods (Fig. 6a). The observation of ΔTEC (Fig. 6b), the simulations of differential TEC (Fig. 6d), and ΣO/N_2_ (Fig. 6c) with similar time and coverage are also displayed. On May 11, the GOLDΣO/N2 indicated an extreme reduction moving from high to low latitudes, even crossing the equatorial region within the 60°W–80°W longitude zone. The intensified ΣO/N_2_ regions began to recede toward higher latitudes in the southern hemisphere after 12:00UT on May 11. On May 12, the reduction of ΣO/N_2_ still moved to low-latitude regions in some longitudinal sectors near sunset time and was mainly located at the region above 30°N in the northern hemisphere, whereas the ΣO/N_2_ in the southern hemisphere presented a slight enhancement. The difference of the absolute TEC (ΔTEC) between the southern and northern hemispheres was consistent with that in ΣO/N_2_. The simulations of ΣO/N_2_ from the TIEGCM were generally in agreement with GOLD's observations, with the depleted compositional disturbance region extending to extreme low latitudes in both the northern and southern hemispheres on May 11, and an enhancement belt in the equatorial region. However, the reduction of ΣO/N_2_ weakened and exhibited enhancement in low latitudes on May 12, which was not consistent with observations in the northern hemisphere. In low-latitude regions the influence of the disturbed electric field may be additionally superimposed, which ultimately leads to the discrepancies between the simulated TEC and the observed TEC.

The change in ΣO/N_2_ well explained the hemispheric asymmetry of TEC, and was one of the reasons for the difference between simulations and observations. Another factor for lower electron density in TIEGCM may be related to the integration height of the TEC in the TIEGCM, which varies at different latitudes but generally does not exceed 1000 kilometers. In contrast, the observed TEC was measured up to the altitude of the GNSS satellites, which is ∼20 000 kilometers. Changes in the topside ionosphere may be one of the reasons for the inaccuracy of the TEC simulated by the TIEGCM.

This study investigated the ionospheric response to the super geomagnetic storm on May 10–12, 2024 based on multi-instrumental measurements from local and global observations. Significant ionospheric negative disturbance expanded from high to low latitudes, covering the whole northern hemisphere and persisting for more than 2 days. The electron density showed an extreme decrease (with a maximum reduction of 98%), causing the northern crest of the EIA to be significantly suppressed on May 11 and almost vanished on May 12, 2024. The observation of vertical drift and ΣO/N_2_ revealed the extreme depletion of ionospheric electron density was mainly caused by the neutral composition disturbance transferred from high latitudes, accompanied by the westward electric field effect driven by PEFs or DDEFs at low latitudes. There was a significant asymmetry in global ionospheric electron density response between the northern and southern hemispheres. The composition observation from GOLD and simulation indicated that the seasonal neutral wind and distribution of O/N₂ may be responsible for the hemispheric asymmetry in ionospheric response.

This study provides valuable observational insights into the ionospheric response during storms and contributes to the understanding of the ionospheric response mechanism at low- and mid-latitudes. The extreme reduction of electron density resulted in the complete disappearance of ionospheric radio echoes at multiple ionosonde stations in a large area over an extended time period. This disruption highlights the significant impact of ionospheric disturbances on high-frequency radio waves.

## METHODS

### Ionosonde data and the identification of radio interruption

The ionogram represents the frequencies of the transmitted radio signals varying with the virtual heights of ionospheric layers. In this paper, the ionograms at 14 ionosonde stations from CMP are used for determining real-time HF radio propagation interruption and absorption properties. The extreme reduction of ionospheric density during a geomagnetic storm sometimes leads to no transmission path and missing HF echoes from ionosondes. Therefore, the interruption induced by a negative ionospheric storm occurs after a storm, possibly during daytime or night-time. During a strong solar flare, the increased X-ray flux and extreme ultraviolet (EUV) radiation penetrates to the D-layer of the ionosphere, and ionizes the atmosphere therein to an enhanced D-layer, which will absorb higher frequencies and disrupt HF radio communication on the sunlit side. The absorption means that the traces at lower ionosphere are obscured, or even disappear completely on the ionogram. The absorption is associated with a flare event and occurs mainly during daytime. According to the sequential variation of ionograms, we evaluate the status of each ionogram one by one and identify the absorption as being caused by solar flare or the interruption by ionospheric negative effects.

### TEC map in the Chinese region

TEC is a widely used parameter in ionospheric investigation and modeling, and can be derived from GNSS dual-frequency observation. The RINEX observation files from 27 GNSS receivers in the CMP were collected and processed into standard daily files. The daily RINEX file from the other 58 GNSS receivers from International GNSS services were also used. To obtain the TEC map for the whole Chinese region, we applied data assimilation methods, combining the observed GNSS-TEC with the International Reference Ionospheric (IRI) model [45] based on the Kalman filtering technique, as described in Aa *et al.* [27]. The final TEC map is 1° × 1° of geographic latitude and longitude bin at a time resolution of 15 minutes. The TEC map was very consistent with the TEC obtained from the Madrigal database (Fig. S4).

### The profile of electron density and ion drift data

The incoherent scatter radar in Sanya (SYISR) is a newly built instrument in the CMP, which can observe the ionospheric plasma density, temperature, and ion velocity [30,46] and subsequently the ionospheric electric field and neutral wind. The electron density and ion drift observations used are the Level 2 product of the SYISR.

## Supplementary Material

nwaf307_Supplemental_File

## Data Availability

The interplanetary magnetic field and solar wind data were sourced from ACE data available at https://sohoftp.nascom.nasa.gov/sdb/goes/ace/daily. The Dst and Kp data are from WDC-2 at Kyoto (http://wdc.kugi.kyoto-u.ac.jp). The TIMED/GUVI data were provided by the Johns Hopkins University Applied Physics Laboratory (http://guvitimed.jhuapl.edu). The GNSS-TEC, ionosonde Ionogram, and electron density and ion velocity measured at SYISR are from https://doi.org/10.57760/sciencedb.space.01964. The global GPS TEC data and the ion density from DMSP were obtained from the Madrigal database at CEDAR (http://cedar.openmadrigal.org). GOLD Level 2 data used in this study are available at the GOLD Science Data Center (https://gold.cs.ucf.edu/search/). Swarm data are provided by the European Space Agency (https://swarm‐diss.eo.esa.int/).
