# Supplementary material for: The extreme depletion of ionospheric electron density and its hemispheric asymmetry during the May 2024 storm
Source: Natl Sci Rev. 2025 Aug 1;12(10):nwaf307. doi: 10.1093/nsr/nwaf307 (PMC12587155; doi:10.1093/nsr/nwaf307)
Supplement: nwaf307_Supplemental_File [file nwaf307_supplemental_file.docx]

**Supplementary Data to**

The extreme depletion of ionospheric electron density and its hemispheric asymmetry during the May 2024 Storm

Yanhong Chen^1†^, Ercha Aa^1†^, Tianjiao Yuan^1,2^, Shunrong Zhang^3^, Hua Shen^1^, Xinan Yue^4^, Heng Xu^1,2^, Siwei Liu^1,2^, Xin Wang^1^, Wengeng Huang^1^, Hui Li^1,2^, Bingxian Luo^1,2*^, Qinghe Zhang^1,2*^, Chi Wang^1,2*†^

^1^Key Laboratory of Solar Activity and Space Weather, National Space Science Center, Chinese Academy of Sciences, Beijing, 100190, China.

^2^University of Chinese Academy of Sciences, Beijing, 101408, China.

^3^MIT Haystack Observatory, Westford, Massachusetts, 01886, USA.

^4^Key Laboratory of Earth and Planetary Physics, Institute of Geology and Geophysics, Chinese Academy of Sciences, Beijing, 100029, China.

†These authors contributed equally to this work.

*Corresponding author. Email: cw@swl.ac.cn, qhzhang@spaceweather.ac.cn, [luobx@nssc.ac.cn](mailto:Luobx@nssc.ac.cn)

Supplementary Data Figures：

Figure S1…………………………………………………………………………….… Page S2

Figure S2………………………………………………...……………………… .....…. Page S3 Figure S3………………………………………………...……………………………. . Page S4

Figure S4………………………………………………...…………… ……….....…. Page S5


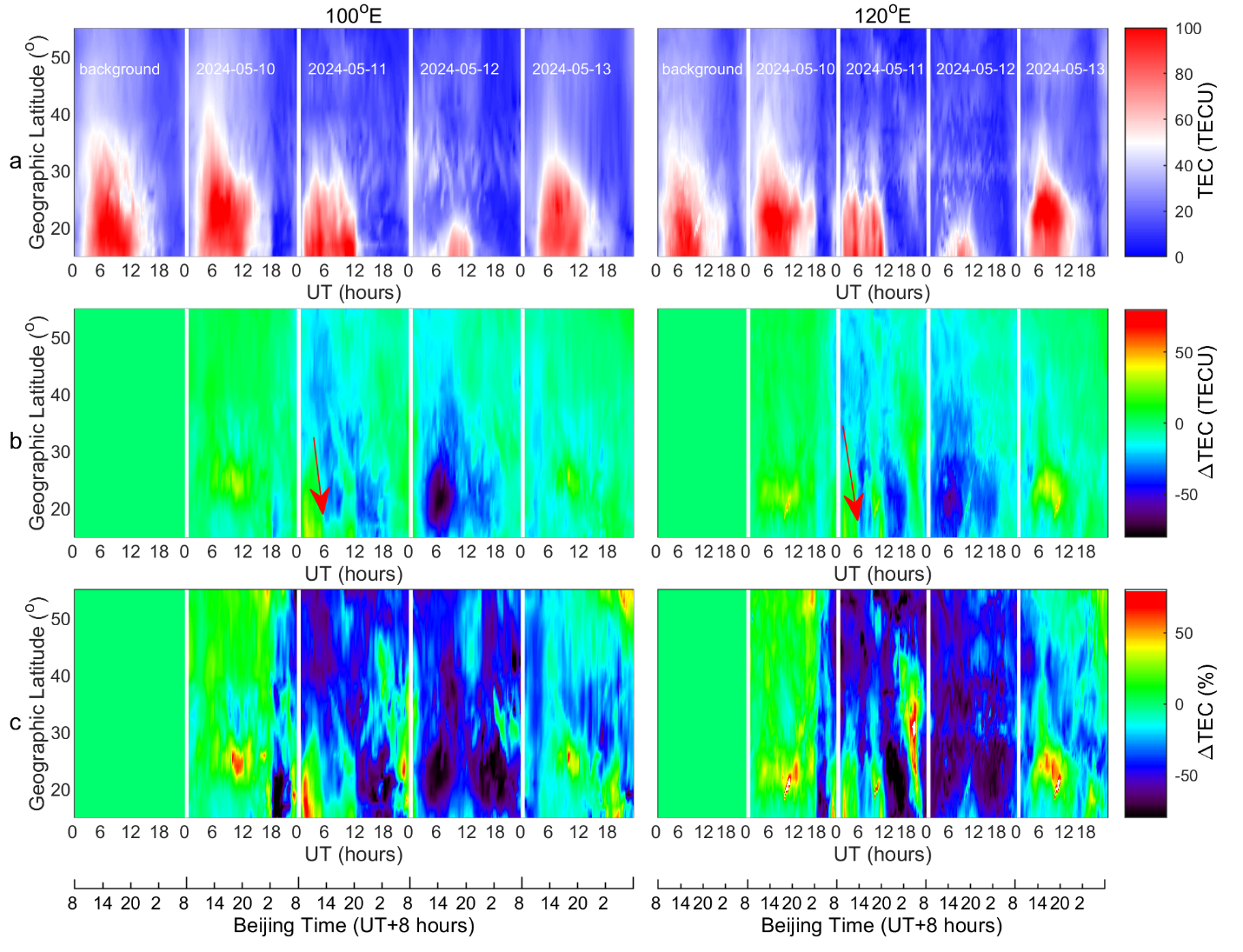


Figure S1. The variations of TEC and the deviation as a function of UT time and Beijing time (UT+8hours) on May 10-13, 2024. The differential TEC is calculated by subtracting the quiet-time TEC, derived from the median values of the assimilation results on 7-9 May, 2024, from the storm-time TEC (a): The background TEC from May 7-9 and the TEC on May 10-13,2024. (b): The differential TEC at longitudes of 100^o^E and 120 ^o^E. The red arrow indicates the expansion of negative disturbance. (c): The relative TEC difference at longitudes of 100^o^E and 120 ^o^E.


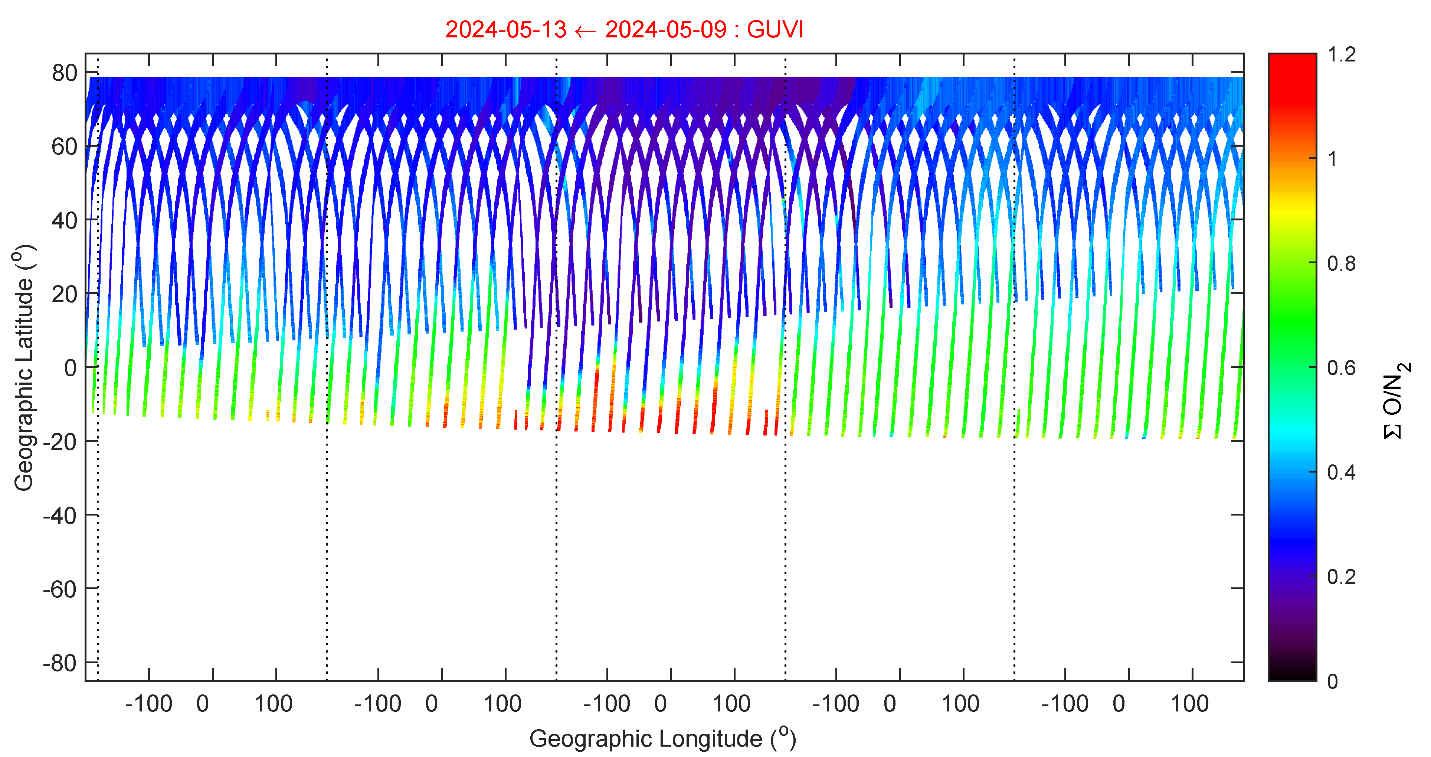


Figure S2. The the column integrated ratio of O and N2 observed by TIMED/GUVI.


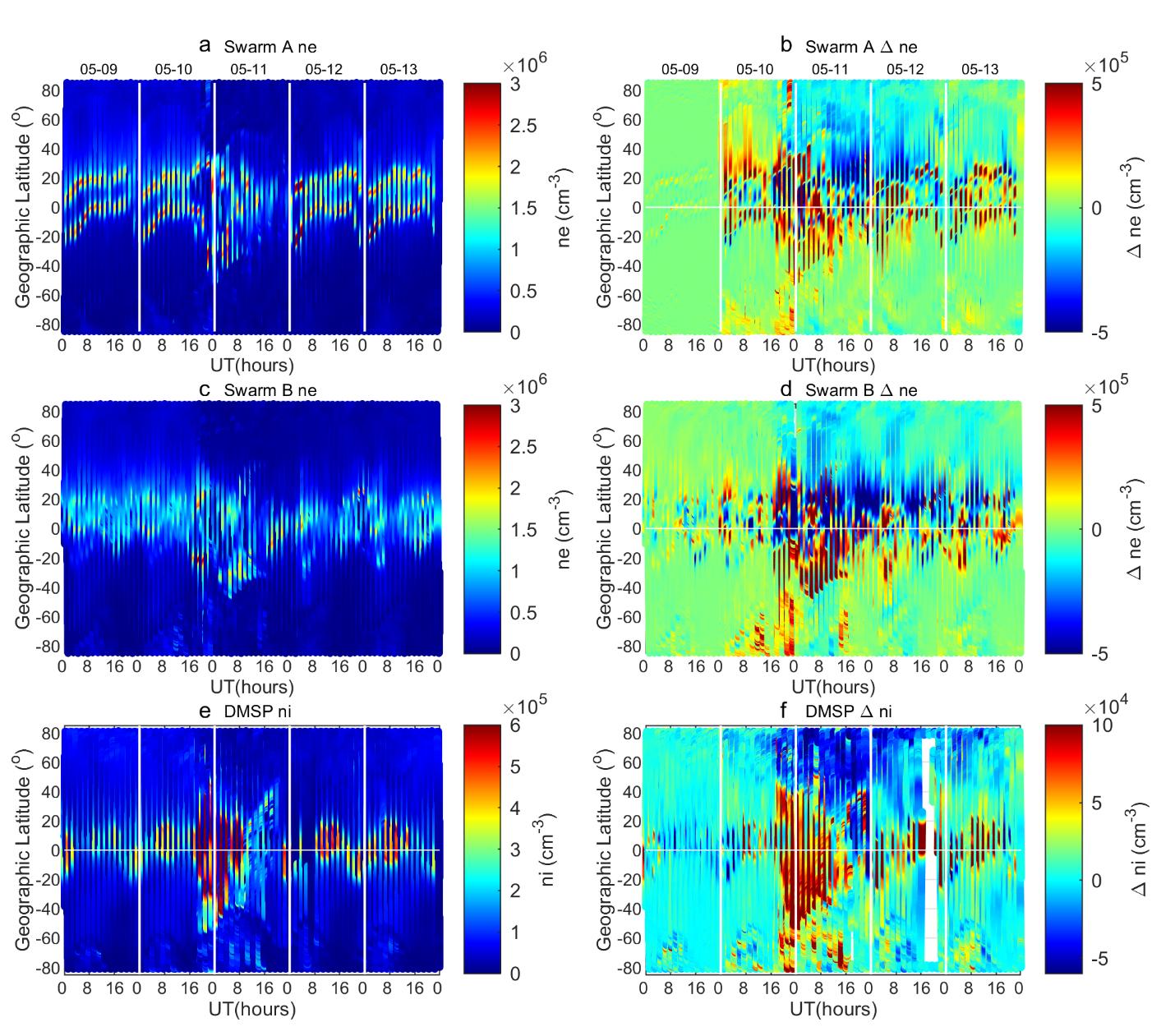


Figure S3. Latitude‐time variations of electron density and plasma density distribution during 9–13 May from Swarm A, Swarm B, and DMSP. (a)-(b) the electron density and the deviation from Swarm A. (c)-(d) the electron density and the deviation from Swarm B. (e)-(f) the plasma density and the deviation from DMSP F16, F17 and F18


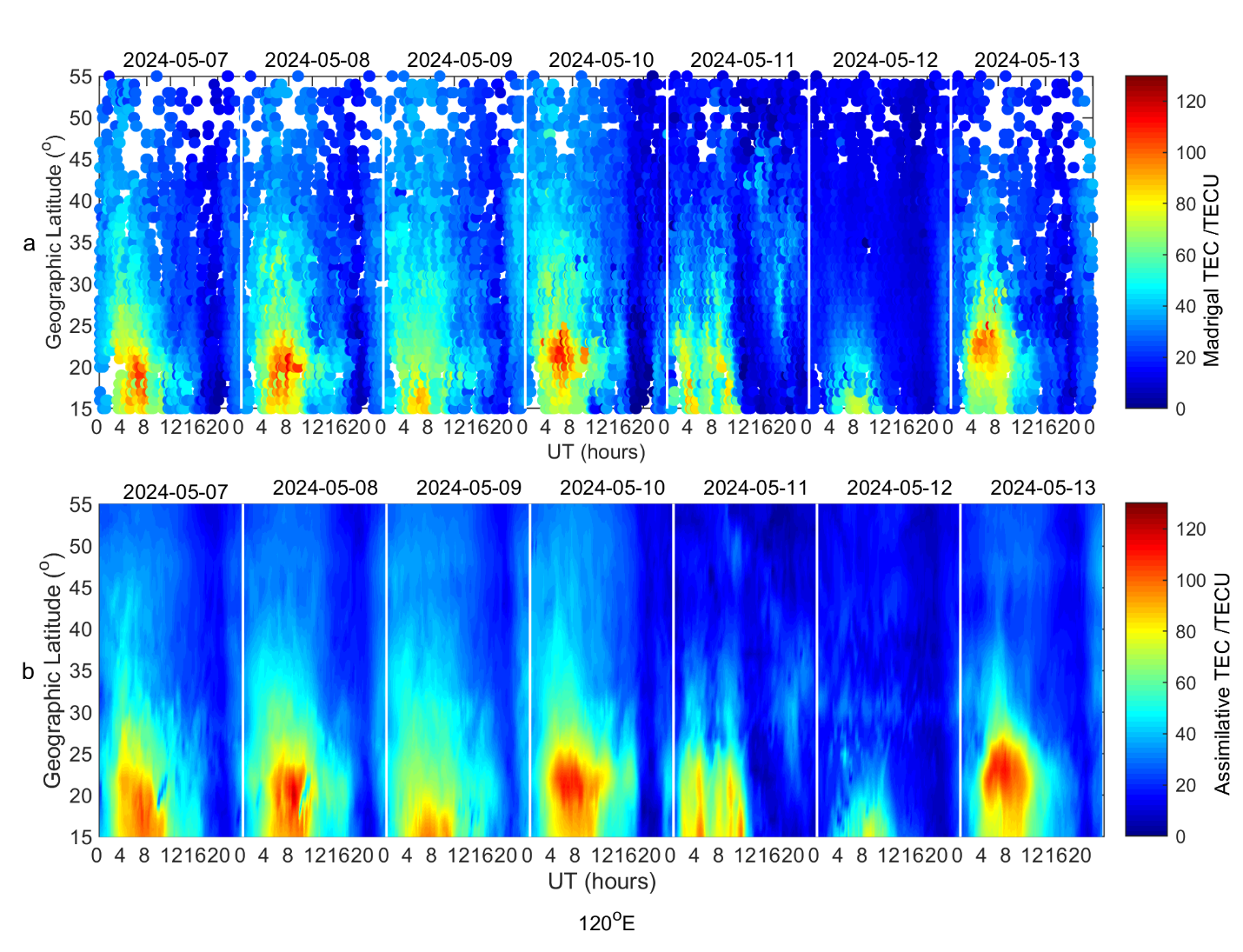


Figure S4. The comparison of madrigal TEC and assimilated TEC in the longitude of 120^o^E during May 2024 Storm. (a) The madrigal TEC from GNSS observations (b) the assimilated TEC map based on CMP observation.
